# *“We don’t know schistosomiasis can affect the female reproductive system and increase the risk of HIV”*: A mixed-methods analysis reveals gaps in risk factor determinants and health consequences of female genital schistosomiasis in Southern Chad

**DOI:** 10.1371/journal.pntd.0014791

**Published:** 2026-10-06

**Authors:** Melissa N. Asongha, Toussaint N. Ngakia, Pascaline Akawung, Chrisvalin Lokissidingao, Dum-Buo Nnamdi, Barou O. Zephanias, Fotsa Blondeau, Abdoulaye Djelamde, David A. Lewis, Efuetacha Betehndoh, Francis N. Nkemngo

**Affiliations:** 1 Centre for Infection Biology and Tropical Health (CIBITH), Buea, Cameroon; 2 Department of Public Health and Hygiene, University of Buea, Buea, Cameroon; 3 Universite de N’Djamena, N’Djamena, Chad; 4 Regional Delegation of Public Health, Mayo-Kebbi West, Chad; 5 Centre for Research in Infectious Diseases, Yaoundé, Cameroon; 6 School of Public Health, University of Alberta, Edmonton, Alberta, Canada; 7 Sydney Medical School-Westmead, Faculty of Medicine and Health, University of Sydney, Sydney, Australia; 8 Division of Medical Virology, Faculty of Health Sciences, University of Cape Town, Cape Town, South Africa; 9 Western Sydney Sexual Health Centre, Western Sydney Local Health District, Parramatta, Australia; 10 Mid Yorkshire Teaching NHS Trust, Wakefield, United Kingdom; Universite de Montreal, CANADA

## Abstract

**Background:**

Urogenital schistosomiasis caused by *Schistosoma haematobium* infection is a public health problem in Chad. Despite reports of urinary *S. haematobium* prevalence in endemic communities, research on the genital aspect of the disease, which disproportionately affects women, remains neglected especially given the contemporary geographical overlap with HIV/AIDS. This study was undertaken to establish baseline data on the awareness and perception of female genital schistosomiasis (FGS) among community inhabitants and healthcare professionals in southern Chad to inform effective policy decision-making.

**Methods:**

A mixed-methods cross-sectional design was employed for data collection in communities within the Guelo district from June to July 2025. Structured questionnaires were applied to capture quantitative data, while qualitative information was obtained through eleven focus group discussions (FGDs) and fifteen key informant interviews (KIIs). Quantitative and qualitative data were analyzed using statistical tools and NVIVO software, respectively, and outcomes were organized according to thematic categories.

**Results:**

A marked disparity characterized by sufficient knowledge about the urinary form of schistosomiasis [93.8% (CI: 91.5 – 96.2), 514/548] and minimal knowledge of FGS [3.1% (CI: 2.3 – 4.9), 17/548] was documented among the population. Moderate [63.2% (CI: 59.4 – 71.7), 325/514] and low [17.7% (CI: 11.3 – 21.8), 3/17] levels of awareness were respectively identified as predictors of urinary schistosomiasis and FGS, consistent with the perception that FGS is neither a risk factor for HIV nor cervical cancer. Additionally, verbal reports among informed community members and healthcare professionals revealed a shared belief that FGS is sexually transmitted and typically treated with antibiotics/antifungal medications. This belief was associated [χ^2^ = 13.7 (CI: 10.1 – 15. 6), *p* = 0.002] with negative consequences on hospital-treatment-seeking habits that arise from fear of identity-related stigma, particularly in cases involving recurrent genital symptoms.

**Conclusion:**

This study highlights the need for integrating FGS education and awareness initiatives within praziquantel delivery campaigns and sexual/reproductive preventive services at primary health care facilities in rural Chad. Furthermore, it underscores the urgency of providing comprehensive training for local healthcare professionals on the diagnosis and clinical management of FGS and the improvement of water, sanitation and hygiene (WaSH) services to minimize contact with freshwater bodies.

## Author summary

Female genital schistosomiasis is a chronic infection affecting adolescent girls and women, particularly in rural communities located near snail-infested river water bodies. Understanding the knowledge and perception of FGS among the local population is key to designing effective interventions. Using a combination of structured questionnaire, FGD and KII, we comprehensively captured a broad range of perspectives on urogenital schistosomiasis, with particular emphasis on FGS and its associated risk factors in southern Chad. Our data revealed limited knowledge and awareness levels characterized by misconception of FGS being a sexually transmitted infection (STI). The lack of awareness was compounded by the overlapping clinical presentations with common STIs, which are often associated with social stigma. Furthermore, verbal responses from local participants indicated that persistent symptoms associated with FGS led to negative hospital health-seeking behaviors. These behaviors were shaped by frequent antibiotic mis-prescriptions from healthcare professionals, which often did not alleviate symptoms, leading to diminished trust in the healthcare system. Overall, this study represents an initial effort in tackling the underrecognized impact of FGS in rural Chad and recommends training of local healthcare professionals on diagnosis and treatment of the condition, as well as implementing culturally appropriate messaging to address knowledge gap about FGS and improvement of WaSH infrastructures to limit water exposure.

## Introduction

Urogenital schistosomiasis (UGS), caused by *Schistosoma haematobium* is a neglected tropical disease that poses a major public health problem in Central Africa [1]. The disease is particularly prevalent in communities that lack sufficient access to water and sanitation facilities [1,2]. Transmission occurs when humans encounter freshwater bodies containing hatched parasite eggs that progress through multiple stages to become cercariae, the infective stage [1]. In Chad, UGS accounted for about 5 million cases in 2021 [3], although regular annual data on the disease burden remains limited. The current approach to UGS control in the country relies on preventive chemotherapy, primarily consisting of periodic administration of praziquantel to school-age children through school-based programs [4,5]. While this strategy effectively reduces morbidity, it does not prevent reinfection and overlooks other exposed groups, such as women and fishermen, who may experience severe reproductive health outcomes [6].

Chronic infection with *S. haematobium* in women can lead to female genital schistosomiasis (FGS), which is marked by the presence of schistosome eggs and distinct morphological abnormalities in the female reproductive organs [7,8]. Eggs trapped in the urogenital tissues induce an inflammatory response, leading to various lesions in the genital tract of women and girls and associated symptoms, including vaginal itching, abnormal discharge, bleeding, and painful intercourse [8,9]. These non-specific symptoms are frequently misdiagnosed as STI-related and can result in inappropriate treatment that drives persistent infection [9,10]. The consequences of misdiagnosis can lead to stigma and depression among affected individuals, especially in rural Chad, where the absence of standard diagnostic equipment, skilled healthcare workforce, and preventive services makes clinical management of the disease challenging [7]. Moreover, genital manifestations of schistosomiasis have been comparatively neglected in contrast to urinary and intestinal manifestations at the health system level [6,11], notably as preventive chemotherapy programs are restricted to school children [12,13], further amplifying the risk of HIV acquisition and cervical cancer among FGS-affected individuals [8,14,15]. Indeed, previous studies have shown that large-scale treatment programs for UGS may have a double impact on indirectly lowering the incidence of HIV, especially in regions where both diseases are prevalent [16,17].

In this context, designing and implementing an effective intervention aimed at clinical management and/or reducing exposure to and risk of acquiring FGS necessitates a comprehensive understanding of the knowledge, attitude, and perception (KAP) of the disease among endemic populations [18]. This is particularly important given the lack of data on the socio-ecological determinants of FGS, including disease perception, health-seeking behavior, and access to treatment in schistosomiasis-endemic communities in Chad. Against this backdrop, this study was undertaken to identify the risk-factor determinants and health-system challenges regarding FGS, with a focus on gathering bottom-top recommendations for advocating awareness among girls and women of reproductive age and capture community opinion on the relevance of expanding preventive chemotherapy intervention beyond school-age children in rural Chad.

## Methods

### Ethics statement

The study received ethical approval (N˚0250/MSP/PMKO/DPSPMKO/2024) from the Provincial Committee on Research Ethics for Human Health of the Mayo Kebbi-West Provincial Delegation of Public Health, Chad. Verbal and written consent was obtained from each participant. Individuals under 18 years provided written assent while consent was obtained from their parents or guardians. The purpose of the study was explained to local authorities and community members. Adherence to confidentiality was upheld, and all data were de-identified using a numerical coding system.

### Study area and sample size

The study was undertaken in the Sahelian southern Region of Chad, specifically within the Guelo district of the Mayo-Kebbi West subdivision. Three communities within the Guelo district (Fig 1), including Poudoue (9.5619°N, 14.327°E), Guetalet (9.6397°N, 14.336°E), and Mourchanne (9.6134°N, 14.3146°E) were purposively selected based on close geographical proximity to the lake, prior schistosomiasis prevalence data ranging between 50% - 55% [3] and history of PZQ school-based preventive chemotherapy, usually implemented annually with >80% treatment coverage in highly endemic – high risk villages [3]. The primary occupations of the local population include subsistence agriculture, cattle husbandry, and fishing activities that depend on water resources such as the rivers (“Mayo”) and Lake Lere. Also, borehole systems are widely utilized by many households as a water source. The population is diverse, with various tribal ethnic groups speaking local dialects such as Moundang and Fulfude [19]. The population is predominantly Muslim, with Arabic and French as official languages. The communities are characterized by insufficient access to potable water and limited hygiene and sanitation infrastructures. Notably, communities exhibited major gaps in WASH indicators: poor latrine access, absence of sewage systems, reliance on well and lake water, lack of hand hygiene facilities and public latrines, and reports of open defecation — further contributing to schistosomiasis transmission. In addition, each community is served by one government-funded public health centre, and a few private clinics offering limited-quality services. Sample size was determined based on the mean prevalence of urogenital schistosomiasis in the area [3] using n = Z^2^ x p (1-p)/e^2^; where Z = 1.96, p = prevalence of urogenital schistosomiasis = 0.55%, and e = error rate = 0.05. Calculating this statistic yields a sample size of 384. However, to ensure adequate sample representation and accommodate for possible voluntary withdrawal and attrition, the original sample size was increased by fifty percent, resulting in a total of 576 participants.

**Fig 1:**
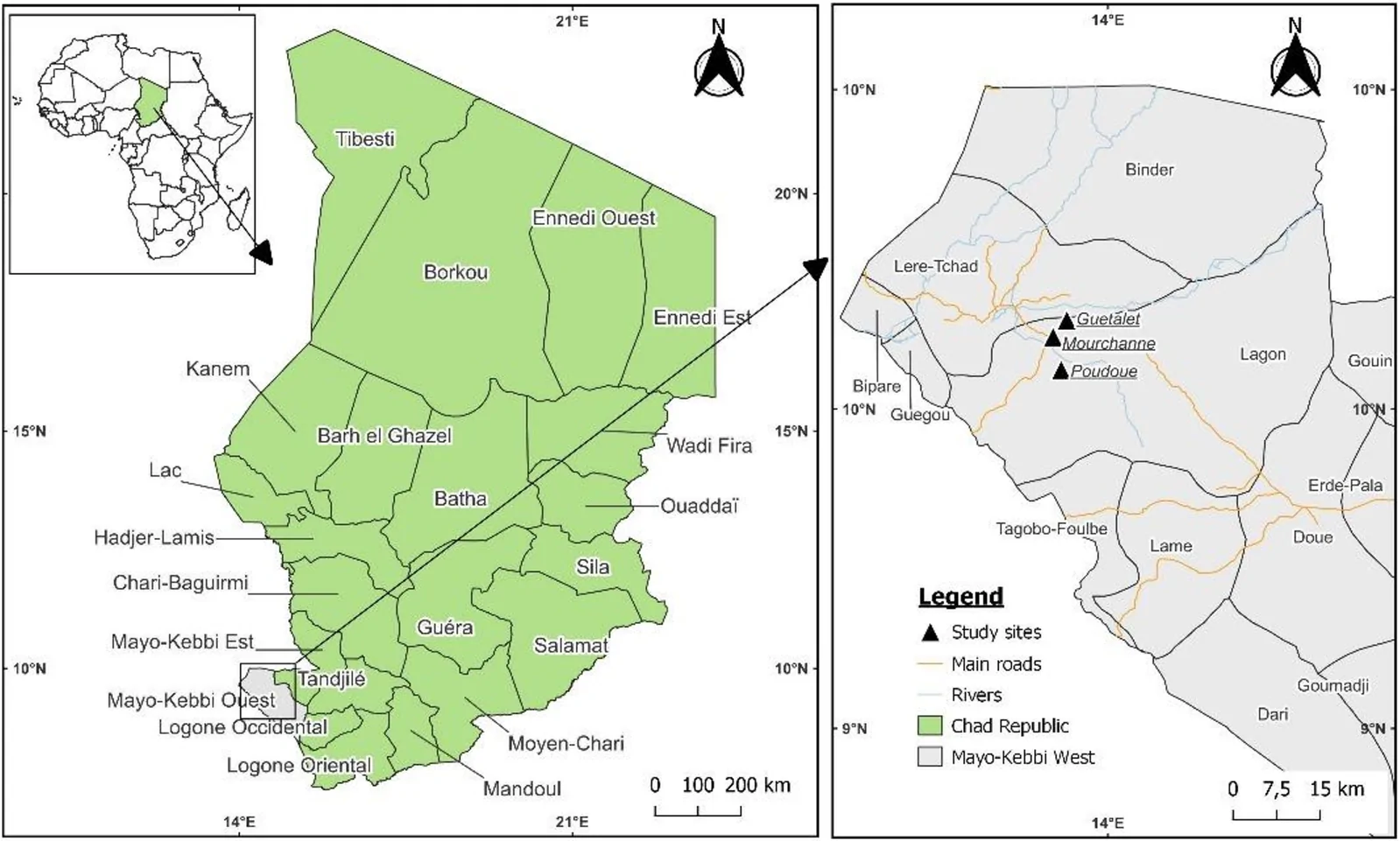
Map of Chad depicting the study localities. Administrative boundaries were obtained from GADM v4.1 and visualized using QGIS v3.40. Country boundary and administrative boundaries shape file of Chad, retrieved from Administrative Boundaries Reference: https://gadm.org/download_country.html and https://gadm.org/maps/TCD.html under an open license (CC BY 4.0): https://gadm.org/license.html. Figure created with QGIS software version 3.40, an open-source Geographic Information System (GIS) licensed under the https://qgis.org/download/. QGIS Geographic Information System. Open-Source Geospatial Foundation. Available at: qgis.org (accessed January 6th, 2026).

### Study design and data collection

Community engagement and advocacy meetings were organized at the Poudoue health district, which included members of the research team, local village leaders, the chief of the health centre, and key community elders (Fig 2). The objective of this activity was to promote inclusive community involvement in the design and implementation of the project. Moreover, such advocacy gatherings aimed to foster local ownership and inclusivity, enhance familiarization, facilitate bidirectional communication, build trust, and support mutual learning on the perceptions about FGS. This approach ensured that the perspectives of local communities were adequately integrated into the research outcomes.

**Fig 2:**
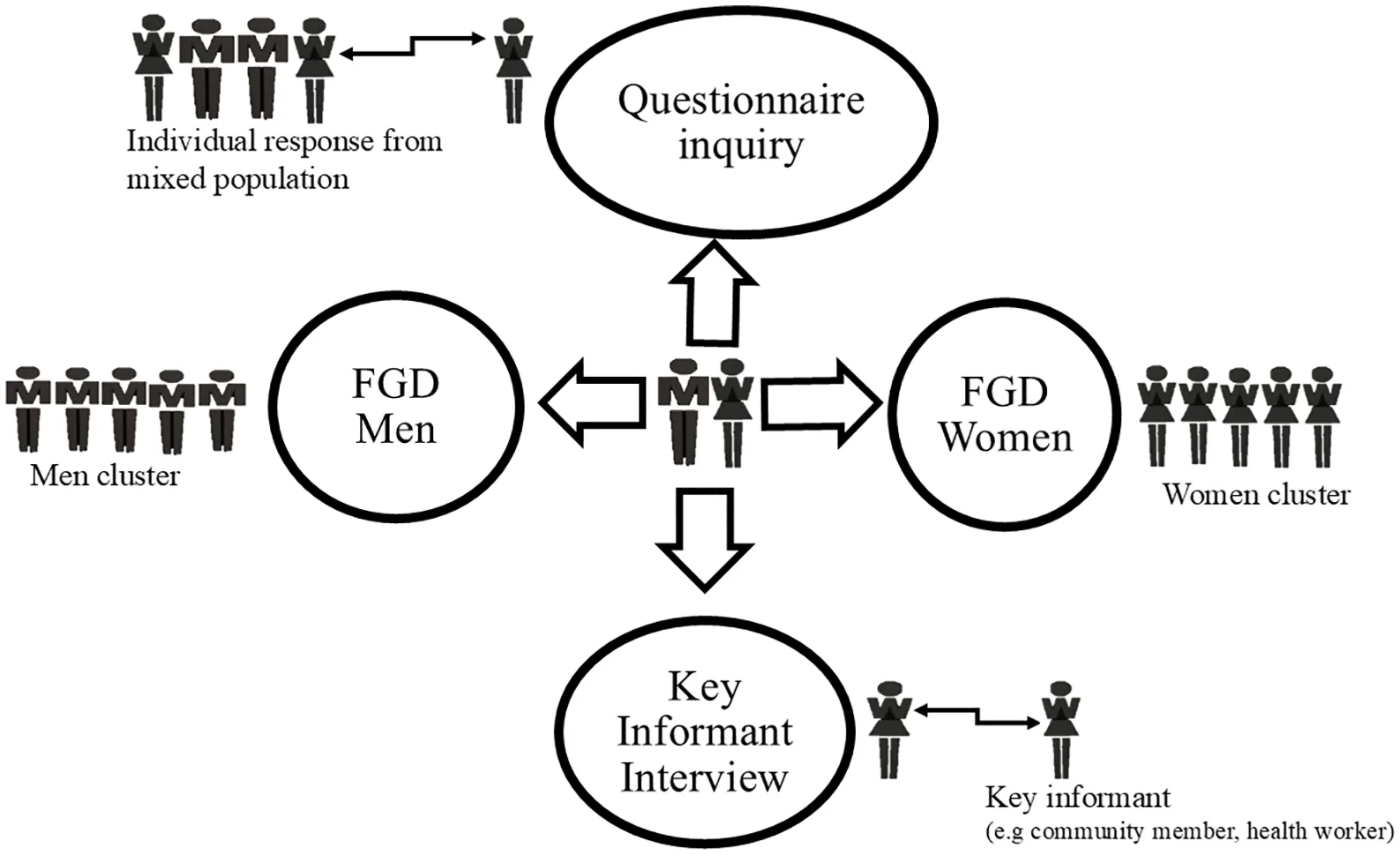
Schematic study design of population sampling and data collection.

This community-based cross-sectional study was conducted between June to July 2025 and employed structured questionnaire, focus group discussions (FGDs), and key-informant interviews (KIIs) for data collection. These qualitative research tools were concurrently used to capture a complete understanding of the sociocultural attributes, beliefs, lived experiences, daily practices, and health system determinants of FGS among inhabitants and health professionals in the community [18,20]. A structured questionnaire (S1 File) was developed to collect variables related to sociodemographic data, etiology, mode of transmission, and treatment for the urinary form of schistosomiasis (hence urinary schistosomiasis) and FGS. The questionnaire gathered responses from participants aged ≥ 8 years who have resided ≥ 12 months in these villages, and included elements on knowledge, symptoms, health risks, and treatment-seeking behaviors specific to FGS. Notably, for children aged 8 – 10 years, administration of sensitive questions about genital health to children followed a parent-guided approach where parents consented and assisted their children in answering the questions properly.

Furthermore, FGDs and KIIs (S2 File) were conducted in the primary local language (Moundang) by four trained research assistants, including the third and fourth authors as well as five experienced community health workers. A total of eleven (11) FGDs (including 04 in Poudoue, 04 in Guetalet and 03 in Mourchanne) were conducted primarily with local community members, each involving young adult females (18–24 years), adult women (25–40 years), elderly women (40 + years), and adult men (25–40 years) in separate sessions. Briefly, each FGD consisted of eight participants and lasted overall 90 minutes [18,21]. The discussions were systematically conducted and audio-recorded, with field notes and daily observation diaries documented by each member to ensure transparency of participants’ views of the subject. Daily diaries were reviewed collectively during recess meetings, and key insights were incorporated into the data analysis process. Due to significant understaffing of healthcare professionals (HPs) in these communities, focus group discussions were not feasible; instead, only key informant interviews were conducted with HPs.

In addition, fifteen (15) KIIs (including 05 in Poudoue, 06 in Guetalet and 04 in Mourchanne) were conducted, and interview participants were purposively selected among local village representatives and health authorities. The selected participants included health centre, staff, auxiliary community health workers, WaSH coordinator, clan elders, and association group leaders. KII was done in a quiet and private setting, and the interaction lasted about 60 minutes to capture maximum information about knowledge scope and health system challenges on urinary and female genital schistosomiasis prevention and control. Before implementation, the questionnaire and interview guide were pretested among 15 randomly selected individuals in a different community to identify potential weaknesses and improve clarity before final administration to the study population.

### Data analysis

Descriptive statistics were employed to assess quantitative relationships between KAP and urinary schistosomiasis/FGS in GraphPad Prism v10. Specifically, Chi-square and Fisher’s exact tests were applied to examine the association between KAP and socio-demographic factors in relation to age and gender. Subsequently, multivariate logistic regression analysis was performed on statistically significant binary outcomes to determine FGS risk factors associated with the KAP variables. Statistical significance was set at *p* < 0.05. For qualitative analysis, digital recordings from FGDs and KIIs were transcribed, and a codebook was subsequently developed using the interview guide. Data integrity for both FGD and KII was maintained through multiple quality control measures, including verbatim transcription to capture subtle details and engaging dual subject-matter experts proficient in both local dialects (Moundang and Fulfude) and bilingual skills for translation. Additionally, translated transcripts and quotations were cross-checked against the original audio recordings and field notes. Furthermore, transcripts were analyzed using a combination of deductive and inductive methods to identify new codes and themes while maintaining their original context. The finalized codebook was used to guide thematic analysis, and transcripts were imported into NVivo 12 software (Lumivero, MA, USA), where related codes were grouped into themes according to identified patterns.

## Results

### Baseline characteristics of the study population

A total of 548 community members participated in the study after filtering for non-response or incomplete response to the questionnaire. Individuals aged 36–45 (29.7%) and females (61.1%) represented the predominant demographic group (Table 1). The participants had a mean age of 33.4 years (ranging from 8 to 65^+^ years), with the majority lacking formal education. Additionally, 11 FGDs were held with community members, and 15 KIIs were undertaken with both community members (n = 8) and healthcare professionals (n = 7) across the three localities.

**Table 1:** Socio‑demographic characteristics of study participants.

Structure	Category	Score, n (%)
Communities	Poudoue	229 (41.8)
	Guetalet	206 (37.6)
	Mourchanne	113 (20.6)
Age (yr)	8–15	94 (17.2)
	16–25	82 (14.9)
	26–35	112 (20.4)
	36–45	163 (29.7)
	>45	97 (17.7)
Gender	Female	335 (61.1)
	Male	213 (38.9)
Educational level	Primary	226 (41.2)
	Secondary	21 (3.8)
	Tertiary	00 (0.0)
	No formal education	301 (54.9)
Occupation	Pupil	51 (9.3)
	Student	43 (7.9)
	Teacher (primary/secondary)	19 (3.5)
	Civil servant	27 (4.9)
	Fishing	152 (27.7)
	Farming	65 (11.9)
	Livestock rearing	110 (20.1)
	Building houses	16 (2.9)
	Petit-trading	39 (7.1)
	Transport service	11 (2.0)
	Unemployed	15 (2.7)

### Knowledge about urinary schistosomiasis and FGS

Analysis indicated that 93.8% (514/548) of participants were aware of urinary schistosomiasis (Fig 3a). The historical awareness of the disease was linked to its familiar clinical symptom (blood in urine), as well as to programmatic annual distribution of PZQ and health prevention information from community health workers. Despite this, a substantial proportion of respondents (Fig 3c) exhibited moderate knowledge regarding the transmission of urinary schistosomiasis, with 36.8% (189/513) incorrectly associating transmission with various factors, including the consumption of dirty drinking water, pork consumption, etc (Table 2). There was no significance between age and gender of respondents with knowledge on FGS [χ^2^ = 0.7 (CI = 0.4 – 0.9), *p* = 0.63] (Fig 3a/3b). Similarly, 96.9% (531/548) of the participants indicated no knowledge of FGS (Fig 3a). The 3.1% (17/548) of participants who reported having heard about FGS cited community health workers as the primary source of information. Of those aware of FGS, 82.4% (14/17) incorrectly identified sexual intercourse as the main cause (Fig 3c), reflecting prevalent misconceptions regarding risk factors and mode of transmission. The remaining respondents who were unaware of FGS expressed uncertainty about whether it is sexually transmitted (Table 2). Additionally, data on common diagnostic tests (Fig 3d) and treatment options (Fig 3d) for FGS-associated symptoms indicate that HIV (57.3%), fungal infection (24.2%), and chlamydial tests (9.6%) are among the most frequently conducted laboratory examinations (Table 2). The primary treatment interventions include antibiotics (57.3%), painkillers (14.6%), and antifungals (21.9%), which are typically prescribed owing to the syndromic overlap between FGS and STIs.

**Table 2:** Age-and gender-stratified knowledge indices regarding urinary schistosomiasis and FGS among the local population.

Urinary schistosomiasis											
Variable		Gender			Age (yr)						χ^2^ [(95%CI), p-value]
		F	M	Total	8–15	16–25	26–35	36–45	>45	Total	
Indicators	Response	Frequency (%)			Frequency (%)						
Have you ever heard of urinary schistosomiasis?	Yes	322 (96.1)	192 (90.1)	514 (93.8)	88 (93.6)	77 (93.9)	104 (92.9)	155 (95.1)	90 (92.8)	514 (93.8)	**65.2** [(53.5 – 71.4) *p* < 0.001*]
	No	13 (3.9)	21 (9.9)	34 (6.2)	6 (6.4)	5 (6.1)	8 (7.1)	8 (4.9)	7 (7.2)	34 (6.2)	
If yes, where did you hear about urinary schistosomiasis?	Radio/TV	39 (12.1)	65 (33.9)	104 (20.2)	5 (5.7)	19 (24.7)	21 (20.2)	40 (25.8)	19 (21.1)	104 (20.2)	**7.3** [(5.1 – 11.6) *p* = 0.02*)]
	School	27 (8.4)	19 (9.9)	46 (8.9)	33 (37.5)	13 (16.8)	0 (0)	0 (0)	0 (0)	46 (8.9)	
	CHW	215 (66.8)	84 (43.8)	299 (58.2)	41 (46.6)	34 (44.2)	69 (66.3)	97 (62.6)	58 (64.5)	299 (58.2)	
	Radio/CHW	46 (14.3)	19 (9.9)	65 (12.7)	9 (10.2)	11 (14.3)	14 (13.5)	18 (11.6)	13 (14.4)	65 (12.7)	
If yes, what causes urinary schistosomiasis?	Bathing in River/Stream	221 (68.6)	104 (54.2)	325 (63.2)	47 (53.4)	53 (68.8)	71 (68.3)	89 (57.4)	65 (72.2)	325 (63.2)	**19.6** [(13.8 – 22.5) *p* < 0.005*]
	Drinking dirty water	47 (14.6)	33 (17.2)	80 (15.6)	16 (18.2)	14 (18.2)	12 (11.5)	27 (17.4)	11 (12.2)	80 (15.6)	
	Pork Consumption	9 (2.8)	6 (3.1)	15 (2.9)	4 (4.6)	0 (0)	1 (1.0)	5 (3.2)	2 (2.2)	15 (2.9)	
	Mosquito bite	2 (0.6)	5 (2.6)	7 (1.4)	3 (3.4)	0 (0)	1 (1.0)	3 (1.9)	0 (0)	7 (1.4)	
	Walking barefoot	19 (5.9)	33 (17.2)	52 (10.1)	10 (11.4)	7 (9.1)	11 (10.6)	19 (12.3)	5 (5.6)	52 (10.1)	
	Bathing in River/Drinking Dirty Water	15 (4.7)	10 (5.2)	25 (4.9)	3 (3.4)	3 (3.9)	5 (4.8)	10 (6.5)	7 (7.8)	25 (4.9)	
	Don’t know	9 (2.8)	1 (3.6)	10 (1.9)	5 (5.7)	0 (0)	3 (2.9)	2 (1.3)	0 (0)	10 (1.9)	
What is your source of daily water use?	Tap water	7 (2.1)	7 (3.3)	14 (2.6)	2 (2.1)	0 (0.0)	1 (0.9)	5 (3.1)	6 (6.2)	14 (2.6)	/
	Well water	145 (43.3)	56 (26.3)	201 (36.7)	31 (33.0)	29 (35.4)	32 (28.6)	45 (27.6)	64 (66.0)	201 (36.7)	
	River/Stream	180 (53.7)	153 (71.8)	333 (60.8)	61 (64.9)	53 (64.6)	79 (70.6)	113 (69.3)	27 (27.8)	333 (60.8)	
**Female Genital Schistosomiasis (FGS)**											
Have you heard of FGS?	Yes	13 (3.9)	4 (1.9)	17 (3.1)	0 (0)	0 (0)	5 (4.5)	9 (5.5)	3 (3.1)	17 (3.1)	**50.3** [(43.4 – 61.7) *p* < 0.001*]
	No	322 (96.1)	209 (98.1)	531 (96.9)	94 (100)	82 (100)	107 (95.5)	154 (94.5)	94 (96.9)	531 (96.9)	
If yes, where did you hear about FGS?	Radio/TV	0 (0)	0 (0)	0 (0)	0 (0)	0 (0)	0 (0)	0 (0)	0 (0)	0 (0)	**61.1** [(55.7 – 69.2) *p* < 0.001*]
	School	0 (0)	0 (0)	0 (0)	0 (0)	0 (0)	0 (0)	0 (0)	0 (0)	0 (0)	
	CHW	13 (76.5)	4 (23.5)	17 (100)	0 (0)	0 (0)	5 (100)	9 (100)	3 (100)	17 (100)	
If yes, what is the cause of FGS?	Drinking dirty water	1 (7.7)	0 (0)	1 (5.9)	0 (0)	0 (0)	1 (20)	0 (0)	0 (0)	1 (5.9)	**27.9** [(23.1 – 35.6) *p* = 0.001*]
	Sexual Intercourse	10 (76.9)	4 (100)	14 (82.4)	0 (0)	0 (0)	4 (80)	7 (77.8)	3 (100)	14 (82.4)	
	Bathing in rivers	2 (15.4)	0 (0)	2 (11.8)	0 (0)	0 (0)	0 (0)	2 (22.2)	0 (0)	2 (11.8)	
	Pork consumption	0 (0)	0 (0)	0 (0)	0 (0)	0 (0)	0 (0)	0 (00	0 (0)	0 (0)	
Is FGS a sexually transmitted infection?	Yes	10 (3.0)	4 (1.9)	14 (2.6)	0 (0)	0 (0)	7 (6.3)	5 (3.1)	2 (2.1)	14 (2.6)	**34.2** [(25.8 – 41.7) *p* = 0.01*]
	No	3 (0.9)	0 (0)	3 (0.5)	0 (0)	0 (0)	0 (0)	2 (1.2)	1 (1.0)	3 (0.5)	
	Unknown	322 (96.1)	209 (98.1)	531 (96.9)	94 (100)	82 (100)	105 (93.8)	156 (95.7)	94 (96.9)	531 (96.9)	
Females: Do you experience the following genital symptoms?	Blood in urine	19 (5.7)	/	/	5 (9.8)	2 (4.4)	0 (0)	5 (5.4)	7 (9.3)	19 (5.7)	/
	Vaginal discharge/itches	47 (14.0)	/	/	2 (3.9)	11 (24.4)	21 (29.6)	9 (9.7)	3 (4)	47 (14.0)	
	Bloody discharge	11 (3.3)	/	/	0 (0)	1 (2.2)	3 (4.2)	5 (5.4)	2 (2.7)	11 (3.3)	
	Bleeding after sexual intercourse	3 (0.9)	/	/	0 (0)	0 (0)	2 (2.8)	1 (1.1)	0 (0)	3 (0.9)	
	Pelvic pain	75 (22.4)	/	/	0 (0)	4 (8.9)	17 (23.9)	39 (41.9)	15 (20)	75 (22.4)	
	Painful urination	19 (5.7)	/	/	7 (13.7)	0 (0)	4 (5.6)	6 (6.5)	2 (2.7)	19 (5.7)	
	None	161 (48.1)	/	/	25 (49.0)	15 (33.3)	9 (12.7)	11 (11.8)	23 (30.7)	161 (48.1)	
Females: What diagnostic test is regularly performed for genital symptoms?	HIV test	102 (57.3)	/	/	2 (3.9)	31 (68.9)	40 (56.3)	22 (23.7)	7 (7.5)	102 (57.3)	**15.3** [(11.7 – 19.8) *p* = 0.02*]
	Fungal (Candida) test	43 (24.2)	/	/	6 (11.8)	11 (24.4)	19 (26.8)	7 (7.5)	1 (1.1)	43 (24.2)	
	Chlamydial test	17 (9.6)	/	/	0 (0)	5 (11.1)	11 (15.5)	1 (1.1)	0 (0)	17 (9.6)	
	Herpes test	0 (0)	/	/	0 (0)	0 (0)	0 (0)	0 (0)	0 (0)	0 (0)	
	Syphilis test	9 (5.1)	/	/	0 (0)	2 (4.4)	6 (8.5)	1 (1.1)	0 (0)	9 (5.1)	
	Vaginal swab	3 (1.7)	/	/	0 (0)	3 (6.7)	0 (0)	0 (0)	0 (0)	3 (1.7)	
	Urine wet mount	0 (0)	/	/	0 (0)	0 (0)	0 (0)	0 (0)	0 (0)	0 (0)	
Females: What treatment do you often receive when you experience these symptoms?	Antibiotic	102 (57.3)	/	/	5 (9.8)	17 (37.8)	43 (60.6)	25 (26.9)	12 (16)	102 (57.3)	**42.6** [(31.7 – 69.3) *p* = 0.001*]
	Painkiller	26 (14.6)	/	/	6 (11.8)	9 (20)	7 (9.9)	1 (1.1)	3 (4)	26 (14.6)	
	Antihelminth	2 (1.1)	/	/	0 (0)	0 (0)	0 (0)	0 (0)	0 (0)	2 (1.1)	
	Antifungal	39 (21.9)	/	/	3 (5.9)	11 (24.4)	15 (21.1)	7 (7.5)	3 (4)	39 (21.9)	
	None	5 (2.8)	/	/	0 (0)	1 (2.2)	1 (1.4)	3 (3.2)	0 (0)	5 (2.8)	

Note: * = Statistical significance at *p* < 0.05 by chi-square test. Where: TV = Television, CHW = Community Health Worker, HIV = Human Immunodeficiency Virus.

**Fig 3:**
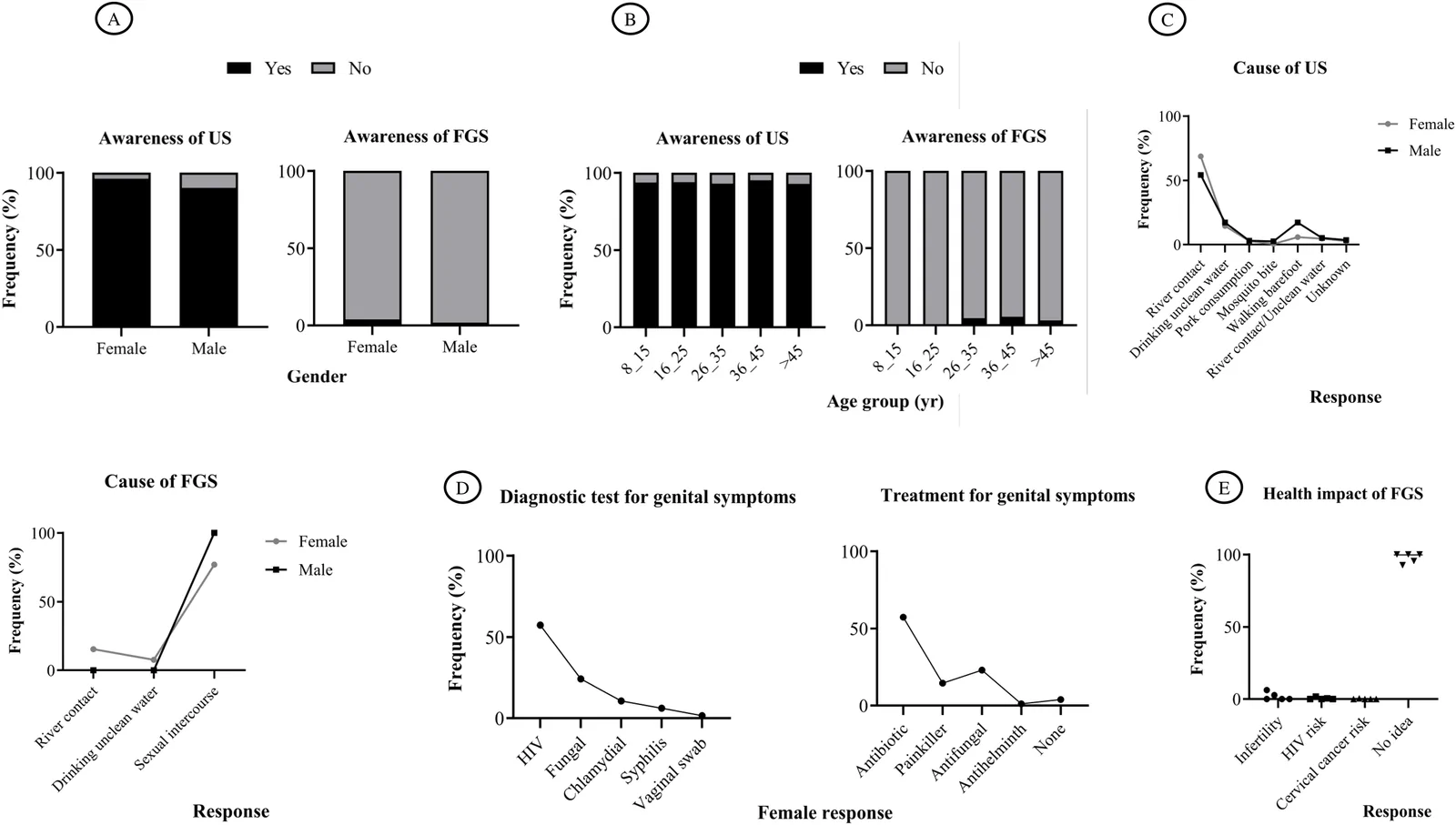
Awareness and perception indicators of urinary schistosomiasis (US) and FGS. **A.** Gender stratified response about the awareness of US/FGS. **B.** Age-group response regarding awareness of US/FGS. **C.** Response of the causes of US/FGS among informed participants. **D.** Documented response of common diagnostic test and treatment prescription for females experiencing abnormal genital symptoms. **E.** Participants’ response on the health impact of FGS. Where US = Urinary schistosomiasis and FGS = Female genital schistosomiasis.

On the other hand, independent focus group discussions conducted with both women and men demonstrated a high level of awareness regarding urinary schistosomiasis. Yet, there was a lack of knowledge regarding its severe effects on the female reproductive system, including the potential to increase the risk of acquiring HIV. Indeed, women participants indicated:

*“”Teutchoum chign” [local dialect meaning of red (bloody) urine] is a common symptom of bilharzia, especially among children and some adults. We know of schistosomiasis [urinary], but we are not aware it can seriously affect the female sexual organs and can increase the risk of having HIV…...Do you mean bathing in the rivers also increases the chances of acquiring HIV?”* Women FGD, Poudoue.

This underscores the disparity in knowledge determinants of urinary schistosomiasis and FGS. Similarly, the women’s FGD further emphasized the limited knowledge of the disease – *“We have heard of schistosomiasis before from the community health worker, but we have no idea what FGS is all about. During mass drug campaigns, health workers always show us poster images of children urinating blood, but none has ever shown images of FGS….. Somehow, FGS is not as important as schistosomiasis [urinary].”* Women FGD, Guetalet. Overall, there was a significant pairwise association [χ^2^ = 21.6 (95% CI: 16.3 – 28.5), *p* = 0.001] between adequate and insufficient knowledge scores on urinary schistosomiasis and FGS, respectively.

### Attitude/practice regarding urinary schistosomiasis and FGS

Although community members demonstrated adequate knowledge of urinary schistosomiasis, their practices remained unchanged. Regular contact with water bodies for domestic and occupational purposes was common practice, with 99.3% (544/548) of respondents reporting routine visits to rivers and indicating willingness to continue such activities despite being aware of the associated risk of infection [χ^2^ = 57.3, (95% CI: 50.6 – 64.1), p < 0.001] (Table 3) especially given the insufficiency of alternative safe portable water supply.

**Table 3:** Attitude and practice scores of urinary schistosomiasis and FGS among study participants.

Urinary schistosomiasis/ Female Genital Schistosomiasis											
Variable		Gender			Age (yr)						χ^2^ [(95% CI), p-value]
		F	M	Total	8–15	16–25	26–35	36–45	>45	Total	
Indicators	Response	Frequency (%)			Frequency (%)						
Do you regularly visit rivers for daily and occupational activities such as swimming, washing clothes, and fishing?	Yes	332 (99.1)	212 (99.5)	544 (99.3)	94 (100)	82 (100)	112 (100)	163 (100)	93 (95.9)	544 (99.3)	**51.7** [(43.2 – 60.5) *p* < 0.001*]
	No	3 (0.9)	1 (0.5)	4 (0.7)	0 (0)	0 (0)	0 (0)	0 (0)	4 (4.1)	4 (0.7)	
Is exposure to river environments associated with the risk of urinary schistosomiasis?	Yes	330 (98.5)	199 (93.4)	529 (96.5)	83 (88.3)	82 (100)	112 (100)	160 (98.2)	92 (94.8)	529 (96.5)	**41.5** [(36.8 – 45.7) *p* = 0.001*]
	No	5 (1.5)	14 (6.6)	19 (3.5)	11 (11.7)	0 (0)	0 (0)	3 (1.8)	5 (5.2)	19 (3.5)	
Would you perform domestic activities (swimming, washing clothes) in these river bodies given the potential risk of contracting urinary schistosomiasis?	Yes	332 (99.1)	213 (100)	545 (99.5)	94 (100)	80 (97.6)	111 (99.1)	163 (100)	97 (100)	545 (99.5)	**57.3** [(54.9 – 61.2) *p* < 0.001*]
	No	3 (0.9)	0 (0)	3 (0.5)	0 (0)	2 (2.4)	1 (0.9)	0 (0)	0 (0)	3 (0.5)	
Is exposure to river environments associated with risk of FGS?	Yes	15 (4.5)	0 (0)	15 (4.5)	0 (0)	0 (0)	11 (9.8)	4 (2.5)	0 (0)	15 (2.7)	**30.6** [(21.7 – 39.4) *p* = 0.007*]
	No	143 (42.7)	193 (57.4)	336 (61.3)	31 (33.0)	51 (62.2)	64 (57.2)	118 (72.4)	72 (74.2)	336 (61.3)	
	No idea	177 (52.8)	20 (9.4)	197 (35.9)	63 (67.0)	31 (37.8)	37 (33.0)	41 (25.2)	25 (25.8)	197 (35.9)	
Females: Would you perform domestic activities (bathing, swimming, washing clothes) in these river bodies given the potential risk of contracting FGS?	Yes	331 (98.8)	/	/	51 (100)	44 (97.8)	68 (95.8)	93 (100)	75 (100)	331 (98.8)	**22.4** [(18.6 – 27.5) *p* < 0.001*]
	No	4 (1.2)	/	/	0 (0)	1 (2.2)	3 (4.2)	0 (0)	0 (0)	4 (1.2)	
Females: Do you experience genital abnormalities (irregular menstruation, itching, foul or bloody discharge, pain)	Frequently	42 (12.5)	/	/	0 (0)	7 (15.6)	19 (26.8)	13 (14.0)	3 (4)	42 (12.5)	/
	Sometimes	62 (18.5)	/	/	0 (0)	16 (35.6)	24 (33.8)	15 (16.1)	7 (9.3)	62 (18.5)	
	Rarely	70 (20.9)	/	/	9 (17.6)	9 (20)	17 (23.9)	25 (26.9)	10 (13.3)	70 (20.9)	
	No	161 (48.1)	/	/	42 (82.4)	14 (31.1)	11 (15.5)	39 (41.9)	55 (73.3)	161 (48.1)	
Females: Where do you often get treatment to relieve genital symptoms?	Hospitals/ clinics	86 (49.4)	/	/	9 (10.5)	19 (22.1)	15 (17.4)	13 (15.1)	2 (2.3)	86 (49.4)	1.1 [(0.4 – 2.3) *p* = 0.08]
	Roadside pharmacy	57 (32.8)	/	/	0 (0)	11 (19.3)	40 (70.2)	29 (50.9)	5 (8.8)	57 (32.8)	
	Tradi-practitioner	31 (17.8)	/	/	0 (0)	2 (6.5)	5 (16.1)	11 (35.5)	13 (41.9)	31 (17.8)	
Females: If you take antibiotics, do the genital symptoms reappear after treatment?	Yes	103 (59.2)	/	/	3 (2.9)	15 (14.6)	46 (44.7)	33 (32.0)	6 (5.8)	103 (59.2)	5.1 [(3.7 – 9.8) *p* = 0.04*]
	No	71 (40.8)	/	/	6 (8.5)	17 (23.9)	14 (19.7)	20 (28.2)	14 (19.7)	71 (40.8)	

Note: * = Statistical significance at *p* < 0.05 by chi-square test.

In contrast, awareness of FGS was markedly limited. While most participants recognized the link between river exposure and urinary schistosomiasis (96.5%), only 2.7% (15/548) identified water contact as a risk factor for FGS, with the majority either reporting against (61.3%, 336/548) or believing no link existed (35.9%, 197/548). This knowledge gap strongly correlated with poor attitudes and preventive practices (r = 0.74, *p* = 0.001). Consequently, 98.8% (331/335) of female respondents reported they would continue performing daily domestic activities in rivers even if such exposure increased the risk of FGS (Table 3).

A considerable proportion of participants (51.9%, 174/335) reported experiencing varying degrees of atypical genital symptoms. Encouragingly, treatment-seeking behavior among these individuals leaned toward formal healthcare: nearly half (49.4%, 86/174) sought care in hospitals or clinics, compared to 17.8% (31/174) consulting traditional doctors and 32.8% (57/174) using roadside pharmacies. Also, 49.4% (86/174) of females expressed a preference for consulting health facility nurses when experiencing genital complaints. However, 59.2% (103/174) reported recurrence of symptom after antibiotic treatment, suggesting possible misdiagnosis or inappropriate treatment of FGS (Table 3).

Furthermore, focus group conversations regarding FGS indicated a uniformity of responses reflecting poor attitude, limited awareness and understanding of the disease. Indeed, respondents stated that:

*“We have been relying on these rivers for decades. We know our rivers can cause diseases like diarrhea and bilharzia, but they remain so important for our daily activities. There is no link between these rivers and FGS……. FGS is caused by sleeping with multiple men without condom; it is sexually transmitted and not by bathing in the rivers we have been using for years. If FGS is caused by the rivers, then all the women would have gotten the disease”* FGD extract from adult women, Mourchanne.

*“I have lived in this community for more than 30 years; you barely find girls urinating blood. Boys are the ones mostly having red urine because you always find them playing and swimming in the rivers. So, I do not think FGS affects women if it is associated with producing red urine*.” KII [F, 53y], Mourchanne.

Considering the rivers as a key source of livelihood, most of the subjects understand that water bodies are involved only in the transmission of urinary schistosomiasis, yet they still engage in river-related activities. This behavioral trade-off, partly due to the community’s reliance on the river for occupational purposes, outweighed concerns regarding the severity of the disease. This was captured in men’s response *– “Most of us are fishermen and solely depend on the rivers to feed our families. We cannot abandon the rivers for a treatable disease like schistosomiasis”* Men FGD, Poudoue

Similarly, discussion among the youthful population emphasized the importance of the river bodies to daily life, particularly given the arid ecological conditions of the region—“*Besides using the rivers to wash clothes, the climate in this village is always very hot, so we regularly visit the rivers to swim and cool off from the heat, especially after returning from work on the farm*.” FGD extract from young adult females, Guetalet.

Likewise, a community inhabitant stated that “*I cannot stop using the river because it is very useful for domestic activities such as washing clothes, watering crops, and as a source of drinking water for my cows.”* KII, F (41y), Poudoue.

These responses underscore the dual role of the rivers: essential for daily life/economic survival yet equally acting as a source for urinary schistosomiasis and potential transmission points for FGS. This reliance on natural water bodies complicates prevention efforts, especially in contexts where safe alternatives are limited.

### Perceptions regarding urinary schistosomiasis and FGS

Neither urinary schistosomiasis nor FGS was regarded as fatal (Table 4). Indeed, about 89% (485/548) of participants quantified the severity of urinary schistosomiasis as mild, based on the symptom of bloody urine. Likewise, due to limited awareness of FGS, both community members and health professionals did not perceive FGS as a serious health concern and equally did not recognize its potential implications for infertility, increased vulnerability to HIV, or increased risk of cervical cancer (Fig 3e).

**Table 4:** Perception indicators predictive of urinary schistosomiasis and FGS among local participants.

Urinary schistosomiasis/ Female Genital Schistosomiasis											
Variable		Gender			Age (yr)						χ^2^ [(95% CI), p-value]
		F	M	Total	8–15	16–25	26–35	36–45	>45	Total	
Indicators	Response	Frequency (%)			Frequency (%)						
Is urinary schistosomiasis a fatal disease?	Yes	45 (13.4)	18 (8.5)	63 (11.5)	7 (13.7)	19 (42.2)	10 (14.1)	12 (12.9)	15 (20)	63 (11.5)	**37.6** [(33.1 – 42.9) *p* = 0.003*]
	No	290 (86.6)	195 (91.5)	485 (88.5)	44 (86.3)	26 (57.8)	61 (85.9)	81 (87.1)	60 (80)	485 (88.5)	
Do you think FGS has severe outcomes?	Yes	13 (3.9)	4 (1.9)	17 (3.1)	0 (0)	1 (1.2)	4 (3.6)	9 (5.5)	3 (3.1)	17 (3.1)	**51.4** [(42.5 – 63.7) *p* < 0.001*]
	No	322 (96.1)	209 (98.1)	531 (96.9)	94 (100)	81 (98.8)	108 (96.4)	154 (94.5)	94 (96.9)	531 (96.9)	
What are the possible health impacts of genital schistosomiasis?	Infertility	9 (2.7)	4 (1.9)	13 (2.4)	0 (0)	0 (0)	3 (2.7)	10 (6.1)	0 (0)	13 (2.4)	**49.7** [(40.3 – 58.6) *p* < 0.001*]
	Increased risk of HIV	3 (0.9)	0 (0)	3 (0.5)	0 (0)	0 (0)	2 (1.8)	1 (0.6)	0 (0)	3 (0.5)	
	Increase risk of cervical cancer	1 (0.3)	0 (0)	1 (0.2)	0 (0)	0 (0)	0 (0)	1 (0.6)	0 (0)	1 (0.2)	
	No idea	322 (96.1)	209 (98.1)	531 (96.9)	94 (100)	82 (100)	107 (95.5)	151 (92.6)	0 (0)	531 (96.9)	
Should praziquantel tablets be given to the whole community?	Yes	335 (100)	213 (100)	548 (100)	94 (100)	82 (100)	112 (100)	163 (100)	97 (100)	548 (100)	**/**
	No	0 (0)	0 (0)	0 (0)	0 (0)	0 (0)	0 (0)	0 (0)	0 (0)	0 (0)	

Note: * = Statistical significance at *p* < 0.05 by chi-square test.

Strikingly, the local population viewed urinary schistosomiasis and FGS as two separate diseases with different etiologies and modes of transmission. FGD assessments from community members generally considered urinary schistosomiasis and FGS to have relatively less severe consequences, which typically do not result in death. Such perception contributes to minimal implementation of effective behavioral prevention strategies against this disease at the community level. This was revealed through common expressions such as:

*“Schistosomiasis is not a deadly disease. It does not kill many people compared to a disease like HIV. If FGS is caused by bacteria that cause schistosomiasis, then it is also not a deadly disease; otherwise, everyone having the disease would have been dead, just like with HIV/AIDS.”* Men FGD*,* Guetalet. This observation highlights the blurred distinction in community perception between urinary schistosomiasis and FGS, with both often regarded as separate and equally less severe conditions.

Interestingly, while both the community inhabitants and healthcare professionals demonstrated sufficient knowledge about urinary schistosomiasis, the majority perceived FGS as a gender-specific disease and had limited understanding of its causative agent and transmission pattern. This was captured in questions such as “*Why does the disease only affect females? Are males resistant to the disease?* FGD, young adult females, Mourchanne and KII, Health professional [M], Poudoue.

*Does the bacteria that causes FGS enter the vagina during bathing or swimming?? Would women have the disease if they do not swim naked in the rivers?* FGD young adult females, Poudoue.

Also, misconceptions about the treatment of FGS among the local health workforce providers were marked by the belief that antibiotics or antifungals were the regimen of choice for genital symptoms due to similar clinical manifestations with common STIs. Indeed, a healthcare professional stated that *“It is difficult to differentiate between FGS and chlamydia. They are all STIs, and doxycycline (antibiotic) is commonly used for treatment. In some cases, women experience recurrent symptoms after the first treatment and are then prescribed a different antibiotic.”* KII extract from healthcare professional [F], Mourchanne. Indeed, such misperception among healthcare providers further obscures the clinical boundaries between FGS and STIs, contributing to misdiagnosis and inappropriate treatment.

Furthermore, community perceptions on FGS treatment-seeking behavior revealed a tendency to seek health clinic consultation upon the onset of genital symptoms. However, the expenses associated with paying for laboratory tests and concerns about symptom-related stigma often led individuals to avoid hospital services and rather purchase medications from unauthorized roadside vendors. This was reflected in several captured responses, including –

*“Visiting the clinic makes the nurses have the impression that you have multiple partners; it creates a stigma, especially as we are all in the same community and see each other every day. Because of this, most young women usually avoid going to the hospital when experiencing itching or vaginal discharge and instead buy medication from roadside sellers.* Women FGD, Poudoue. Similarly, a health care provider mentioned that - *“Most young girls and women don’t prefer coming to the clinic when having recurrent vaginal symptoms because they feel ashamed and stigmatized.”* KII extract from healthcare professional [M], Guetalet.

In cases where symptoms continued despite clinical prescriptions, participants reported a shift in care-seeking behavior from clinics to local traditional practitioners with whom they reported feeling more comfortable. Substantial out-of-pocket expenditure, accessibility to hospital settings, and treatment ineffectiveness were the common complaints. This was documented from a participant saying, *“I prefer seeing a traditional doctor for treatment, because hospital consultation is time-consuming and expensive, and the drugs from the hospital often don’t work—the symptoms usually reappear after I finish taking the drugs (antibiotics) prescribed by the nurse. So, I prefer taking the herbs, and it works better.”* KII extract, community member [F, 36y], Mourchanne.

Regarding the existence of training opportunities on clinical diagnosis and management of genital schistosomiasis, healthcare providers acknowledge that such opportunities are lacking. Indeed, healthcare professionals revealed that “*We participate in district-level trainings focused mainly on diseases such as HIV, Hepatitis B, and vaccine-preventable infections (e.g., cholera, polio, measles) and not FGS”*. KII extract from healthcare professional [M], Mourchanne

*“Community health centres are not equipped to handle reproductive health diseases; the equipments are not available and training is lacking”.* KII from healthcare professional [M], Guetalet

On a positive side, both community members and healthcare professionals expressed similar opinions on the perception regarding extending preventive chemotherapy beyond school children to benefit community-wide treatment of urogenital schistosomiasis.

*“I think praziquantel should be given to the whole community, just like how Mectizan is given. We all use these rivers for washing dresses, bathing, fishing etc; so, everyone is at risk of catching schistosomiasis.”* KII extract from community member [F, 50y], Poudoue.

*“Praziquantel mass drug administration should be provided to everyone in the village, rather than only schoolchildren, as the entire community routinely utilizes the rivers for daily activities and income generation and, as such, gets exposed to acquiring female genital schistosomiasis.”* KII extract from healthcare professional [M], Guetalet.

### Multivariate analysis between socio-demographic factors and KAP regarding FGS

In Table 5, logistic regression analysis revealed no association between age, educational level, and knowledge of FGS [aOR: 0.7 (95% CI: 0.5–1.3), *p* = 0.3]. However, a significant relationship was observed between frequent water contact as potential risk factor and verbal reports on genital symptoms predictive of FGS, particularly for adult women [aOR: 7.3 (95% CI: 4.5 - 11.6), *p* = 0.002]. Additionally, a marked association [aOR: 6.5 (95% CI: 4.1–9.5), *p* = 0.001] was found between limited FGS knowledge and STI differential diagnosis, as reported by both community members and healthcare professional.

**Table 5:** Probable risk factor drivers of FGS based on KAP statistics.

Female genital schistosomiasis			
Variable	Comparator	Crude OR, 95% CI (p value)	Adjusted OR, 95% CI (p value)
Knowledge/awareness of FGS	Age	1.1 [0.4 – 2.1] (0.4)	0.7 [0.5 – 1.3] (0.3)
	Gender		
	Educational level		
	Knowledge of urinary schistosomiasis	**9.6 [5.4 – 14.6]** (0.002*)1	**9.9 [5.3 – 12.1]** (0.002*)
Complaints of genital symptoms	Frequent river activities	**6.1 [4.3 – 10.5]** (0.03*)	**7.3 [4.5 – 11.6]** (0.002*)
	STI clinical diagnosis	**5.8 [3.0 – 12.2]** (0.001*)	**6.5 [4.1 – 9.5]** (0.001*)
	Antibiotic/antifungal treatment	**7.0 [5.3 – 11.8]** (0.01*)	**9.4 [5.7 – 13.6]** (0.01*)
Linkage perception of FGS	HIV/AIDS	**5.5 [3.2 – 18.1]** (0.001*)	**7.3 [4.1 – 15.2]** (0.001*)
	Cervical cancer/Infertility		

Note: OR = Odd Ratio; * = Statistical significance at *p* < 0.05. Where STI = Sexually Transmitted Infection.

## Discussion

Understanding the social and anthropological determinants influencing disease transmission forms the cornerstone of successful urogenital schistosomiasis control efforts in endemic communities [18,22]. This study investigated, for the first time, the knowledge, attitude, and practice (KAP) characteristics regarding FGS among community members and healthcare professionals in a schistosomiasis-endemic district in rural Chad and revealed key insights on the divide between urinary schistosomiasis and FGS. While most of the participants reported having heard about urinary schistosomiasis from local community health providers, their knowledge regarding the etiology and risk factors associated with the disease remained inadequate. Compared to urinary schistosomiasis, awareness of FGS among the local population was limited, and many people attributed its cause to sexual intercourse, possibly due to the term “genital” in its name. This difference can be attributed to the well-known symptom of bloody urine, along with the familiar implementation of praziquantel preventive chemotherapy programs, thereby accounting for higher levels of awareness about urinary schistosomiasis than FGS [20,23]. In contrary, the conflation of urinary schistosomiasis and FGS under the umbrella term “urogenital schistosomiasis” may have obscured local awareness of FGS, as preventive chemotherapy efforts have historically placed greater emphasis on the urinary symptoms of the disease rather than its genital manifestations. Moreover, the absence of functional sexual and reproductive services in these underserved remote primary health centers also contributed to the limited knowledge and awareness about FGS. Furthermore, because the clinical presentation of FGS closely resembles that of other commonly acquired STIs, there is a high tendency of symptom-related awareness masking among health professionals, especially given the lack of FGS clinical training and health education campaigns in these localities. The findings of this study align with observations from Tanzania [18], Cameroon [24], Nigeria [25], Ghana [22,26], and the Democratic Republic of Congo [20], where limited awareness of FGS among local community members was also reported.

Ecological correlates play a significant role in the geographical transmission of urogenital schistosomiasis [27]. Insufficient access to clean water facilities often leads communities to depend on local river bodies for domestic and occupational purposes, which in turn increases exposure to disease transmission [27,28]. This is the situation in the Guelo district, where local populations continue to use communal river water sources, despite being fully aware that this practice is highly associated with an increased risk of contracting urinary schistosomiasis, but not FGS. This routine water-contact habit was further reinforced by the opinion that neither urinary schistosomiasis nor FGS was considered a fatal disease like HIV; as such, infection with the parasitic worm does not lead to death. Interestingly, informed community members did not attribute FGS to riverine activities but rather to an STI. Although a segment of the informed local population considered FGS to be an STI, they did not believe it increased the likelihood of HIV acquisition or linked with cervical cancer. This misconception highlights weaknesses in sensitization and health education opportunities among local healthcare professionals, as seen in similar contexts in Cameroon [29], Ghana [30], and Tanzania [31], further underscoring the need for incorporating FGS-specific photographic messaging in schistosomiasis control efforts at the community level.

Furthermore, as reflected in the survey and interviews, both community members and local healthcare professionals demonstrated minimal knowledge on the cause, transmission pattern, symptoms, and health complications of FGS. Indeed, it is unsurprising that healthcare professionals did not consider FGS as a potential underlying cause of abnormal genital symptoms reported by participants, instead commonly identifying genital abnormalities as presentations of STIs and as a result prescribing antibiotic or antifungals for treatment. This further illustrates how atypical genital symptoms which may be predictive of FGS is frequently considered as STI by both community members and health providers and may result to inappropriate treatment. Moreover, according to local healthcare professionals — the existence of limited training opportunities, combined with the lack of diagnostic services, impede healthcare providers’ understanding of FGS [31]. This results in a gap between healthcare professionals’ knowledge of suspected diagnostic and treatment algorithms and the needs of individuals presenting with atypical genital manifestations. The consequence is mistrust of health service providers, which thus encourages the pursuit of alternative treatments, such as traditional herbal remedies. Similar findings were reported in Ghana [23], Tanzania [31], and Cameroon [29], where community inhabitants preferred consulting traditional folklore treatment practices. Likewise, females who experienced recurrent genital symptoms and sought clinical care were labelled as having multiple sexual partners and diagnosed with STIs. Such perceptions create stigma and negatively shape the health-seeking behavior of affected individuals [18,32]. It is therefore essential to implement cohesive public health initiatives to raise awareness about FGS and its clinical manifestations, provide education on preventive strategies, and establish comprehensive training for the diagnosis and clinical management of the disease at the community level [33,34]. This will encourage young girls and women to seek proper treatment and receive psychological and mental health support from healthcare providers at clinical facilities.

In addition, the impressive views of the local population about scaling up school-based PZQ administration to community-wide treatment highlight the need of providing equal access to all at-risk individuals and ensuring no one is left behind as outlined in the 2030 urogenital schistosomiasis elimination roadmap [4]. Inclusion of girls and women in mass drug administration schemes as well as integration of FGS services into existing HIV/AIDS and reproductive health programs will strengthen communal leadership against the disease. Indeed, tackling FGS in schistosomiasis-endemic communities will require four cardinal elements [7,35], including expanding access to clean water supply and sanitation facilities, implementing community-based screening programs for early detection of genital abnormalities, delivering health education initiatives that emphasize supportive and non-stigmatizing messaging, and ensuring sustainable mass administration of praziquantel.

Although this study provides relevant outcomes, several limitations exist. Firstly, the purposiveness in selecting the communities implies that findings are drawn from the experiences of a population subset within defined socio-demographic attributes and may not comprehensively capture the full spectrum of perspectives within the broader heterogeneous population and across the district regarding the disease. This is in line with the observation of a marginally wide confidence intervals especially regarding the perceived linkage between FGS and reproductive health outcomes (such as HIV/AIDS, cervical cancer etc) indicating greater variability in these specific estimates. Secondly, the qualitative response and narratives of participants may have been influenced by the willingness to participate in the study, particularly given the sensitive nature of the question guides used in the data collection process. Thirdly, given the limited number of health professionals present in these communities, the assessment of health system challenges was largely assessed from the prism of community members than from healthcare providers. Finally, gynecological examinations to determine the actual prevalence of FGS were not performed; instead, existing community schistosomiasis prevalence data was used as a proxy to undertake the current study. Nonetheless, this may not be an accurate indicator as current schistosomiasis prevalence may not reflect current FGS prevalence, given that FGS can persist even after active *S. haematobium* infection has resolved. Despite these limitations, our study reveals a web of limited knowledge, negative prevention attitudes, and misconceptions fueled by a lack of training opportunities for local healthcare professionals and inadequate community health education programs, which all intersect to hinder effective social and behavioral change strategies necessary for preventing urogenital schistosomiasis transmission in these localities.

## Conclusion

The study revealed striking polarization in KAP between urinary schistosomiasis and FGS among populations in rural Chad. Both urinary schistosomiasis and FGS represent distinct manifestations of the same parasitic disease, yet FGS is misunderstood as an STI and not recognized as a potential HIV risk factor — underscoring the need for improving health awareness campaigns during preventive chemotherapy programs in these communities. Further studies to determine FGS prevalence and co-develop community-led solutions to identify ways of improving knowledge on the blurred distinction between urinary schistosomiasis and FGS will be relevant to inform policy decisions.

## Supporting information

S1 File Questionnaire. (DOCX)

S2 File FGD and KII guide. (DOCX)

S3 File Deidentified raw data. (XLSX)
