# Supplementary material for: “We don’t know schistosomiasis can affect the female reproductive system and increase the risk of HIV”: A mixed-methods analysis reveals gaps in risk factor determinants and health consequences of female genital schistosomiasis in Southern Chad
Source: PLoS Negl Trop Dis. 2026 Oct 6;20(10):e0014791. doi: 10.1371/journal.pntd.0014791 (PMC13657329; doi:10.1371/journal.pntd.0014791)
Supplement: Supplementary file 1 [file pntd.0014791.s001.docx]

**Questionnaire (la durée maximale de l'interview est de 10 min)**

Remarques: 1. Prenez toujours les coordonnées GPS

Nom de la communauté: ___________________Code de la communauté: ________ ID ________

Nom du participant _______________________________________ Âge(y):________ Sexe:_______

**Section A: Données sociodémographiques**

T^0^_______ Poids_______ années vécues dans le village______ Tribu___________________

**Profession**: Agriculteur	Pêcheur	Enseignant	Infirmier	Agent de santé	Autres__________

**Éducation**: Primaire	Secondaire	Tétiaire	Professionnel	Aucun_________

**Section B: Schistosomiase (ou bilharziose)**

| Avez-vous déjà entendu parler de Bilharzia | Oui | Non |
| --- | --- | --- |
| Si oui, où avez-vous obtenu les informations sur la bilharziose?  Radio/TV Ecole Agents de santé Communauté Jamais |  |  |
| Quelles sont les causes de la bilharziose (signification de la bilharziose dans la langue locale) Sorcellerie / Esprits maléfiques Aliments acides Se baigner dans les rivières Boire de l'eau sale Élevage de porcs Piqûre de moustique Marcher pieds nus Jamais |  |  |
| Y a-t-il une source d'eau dans votre communauté? | Oui | Non |
| Quel type de source d'eau utilisez-vous pour vos activités quotidiennes Eau du robinet Eau de puits riviere |  |  |
| Vous baignez-vous dans les ruisseaux / rivières? | Oui | Non |
| Ressentez-vous les symptômes suivants  Sang dans les urines Miction douloureuse Sang dans les selles |  |  |
| Ressentez-vous les symptômes suivants  Sang dans les urines Pertes vaginales Écoulement sanglant Saignement après un rapport sexuel Sensation de démangeaison Douleur pelvienne Douleur pendant/après les rapports sexuels Saignements intermenstruels Miction douloureuse Sang dans les selles |  |  |
| Urinez-vous ou déféquez-vous dans les cours d'eau ou riviere de la communauté? | Oui | Non |
| Votre maison a-t-elle des toilettes? | Oui | Non |
| Site préféré que vous excrétez souvent: Latrines à fosse Toilettes à chasse d'eau Buisson Riviere |  |  |
| Avez-vous fréquemment de la diarrhée? | Oui | Non |
| À quelle fréquence recevez-vous un traitement contre les vers?  Deux fois par an Une fois par an Quand je suis malade Jamais |  |  |
| Savez-vous qu'il existe une nouvelle formulation de traitement pour le PZQ pour les enfants (1-4) | Oui | Non |
| Votre école a-t-elle un club de santé? | Oui | Non |

**Section D: Schistosomiase génitale**

| Avez-vous déjà entendu parler de la schistosomiase génitale féminine ou masculine? | Oui | Non |
| --- | --- | --- |
| Si oui, où avez-vous obtenu les informations sur le schistosomiase génitale?  Radio/TV École Agents de santé Communauté Jamais |  |  |
| Qu'est-ce qui cause le schistosomiase génitale? Eau sale Sorcellerie Rapports sexuels Se baigner dans un ruisseau Élevage de porcs Je ne sais pas |  |  |
| Baignez-vous ou lavez-vous souvent vos robes dans les ruisseaux et les rivières? | Oui | Non |
| La schistosomiase génitale est-elle une maladie sexuellement transmissible? | Oui | Non |
| Ressentez-vous les symptômes génitaux suivants?  Sang dans les urines Pertes vaginales Écoulement sanglant Saignement après un rapport sexuel Sensation de démangeaison Douleur pelvienne Douleur pendant/après les rapports sexuels Saignements intermenstruels Miction douloureuse Sang dans les selles |  |  |
| Quel test de diagnostic est régulièrement effectué lorsque vous vous plaignez de symptômes génitaux ? VIH Syphilis Chlamydia Herpès |  |  |
| Quel traitement recevez-vous souvent lorsque vous ressentez ces symptoms?  Antibiotiques anti-douleur antihelminthes antiviral antifongique |  |  |
| Si vous prenez des antibiotiques: Les symptômes disparaissent-ils complètement après le traitement ou réapparaissent-ils ? | Oui | Non |
| Visiter l'infirmière au sujet de symptômes génitaux me met mal à l'aise? | Oui | Non |
| Savez-vous que la schistosomiase génitale peut entrainer?  Infertilité Risque accru de VIH Risque accru de cancer du col de l'utérus |  |  |
| Où obtenez-vous souvent un traitement pour soulager ces symptoms? hôpital herboriste traditionnel chef religieux Pharmacie |  |  |
| Prenez-vous du praziquantel pendant la campagne médicamenteuse de masse contre la schistosomiase? | Oui | Non |
