# Supplementary material for: “We don’t know schistosomiasis can affect the female reproductive system and increase the risk of HIV”: A mixed-methods analysis reveals gaps in risk factor determinants and health consequences of female genital schistosomiasis in Southern Chad
Source: PLoS Negl Trop Dis. 2026 Oct 6;20(10):e0014791. doi: 10.1371/journal.pntd.0014791 (PMC13657329; doi:10.1371/journal.pntd.0014791)
Supplement: Supplementary file 2 [file pntd.0014791.s002.docx]

**INDEPENDENT FOCUS GROUP DISCUSSION (FGD) GUIDE FOR COMMUNITY MEMBERS IN GUELO DISTRICT**

I. **Young adult females + Adult females + Elderly women**

| Date of FGD: | __ __ / __ __ / __ __ __ __ (dd-mmm-yyyy) |
| --- | --- |
|  |  |
| Research Assistants | ___________________________________ |
| FGD number: | ___________________________________ |
| Type of participant | _________ |
| Start time (in 24 hour): | ________-Hours |
| End time (in 24 hour): | _______ - Hours |
| Duration of the interview: | ____: ____ (Minutes: Hours) |
| Total number of participants | __________Females |

***Instruction to Research team:*** *After each question is asked, participants should be given enough time to respond. The lead researcher should feel comfortable allowing periods of silence during discussions, rather than feeling compelled to ask additional questions. The key points should be explored systematically to gather adequate information about the participant’s response, especially when engaging quiet participants.*

1. **What is schistosomiasis?**

- *Ask about the local name(s) for schistosomiasis in the community.*
- *Ask members if they or any family member have ever suffered from schistosomiasis.*

2. **How do you acquire schistosomiasis?**

- *Ask FGD members how schistosomiasis is transmitted.*
- *Ask FGD members about their knowledge of the different forms of schistosomiasis.*
- *Request opinions from members regarding their perspectives on whether schistosomiasis can affect the reproductive organs.*

3. **What are the common symptoms of schistosomiasis?**

- *Ask how to know people who have schistosomiasis in the community.*
- *Ask if schistosomiasis affects children, adult women, and men to the same extent, and what symptoms are associated with the disease.*
- *Ask FGD members if they consider schistosomiasis to be a fatal disease.*

4. **How do you treat schistosomiasis?**

- *Inquire from FGD members about how adults with schistosomiasis access treatment, focusing on (i) community-directed treatment provided by community health workers (CHWs) and (ii) the use of herbal concoctions.*

5. **Do you engage in contact with rivers or streams?**

- *Ask FGD members about river contact frequency.*
- *Inquire about the reason for visiting rivers or streams.*

6. **What is female genital schistosomiasis?**

- *Ask members if they know about FGS.*
- *Where did you obtain the information about FGS?*

7. **What is the cause of FGS?**

- *Explore what the cause of FGS is.*
- *Inquire their opinions on whether FGS can be spread through sexual intercourse.*
- *Inquire whether members believe there is a connection between FGS and visits to community rivers.*

8. **Do you experience genital abnormalities, and how do you treat them?**

- *Inquire about the manifestation of FGS.*
- *Inquire whether members experience symptoms such as genital itching or vaginal discharge and how often.*
- *How do you manage these symptoms? Do you seek treatment at the health centre or other facility?*
- *When you seek treatment at a health centre, what diagnosis does the nurse provide?*
- *Inquire about the type of medications given to alleviate genital symptoms.*
- *Was the medication administered by the nurse effective in alleviating the genital symptoms? (i.e. was the treatment effective?)*
- *Did the genital symptoms reappear after completion of treatment?*
- *If yes, did you return to the health center for a follow-up consultation?*
- *If you did not return to the health facility, did you seek an alternative treatment option?*
- *Where do you currently seek treatment when you experience abnormal genital symptoms?*
- *How comfortable are you with visiting the health facility when experiencing genital itching/discharge? Please explain your response.*

9. **How severe do you think FGS is?**

- *Ask members whether they would consider genital symptoms associated with schistosomiasis as a serious health condition.*
- *Probe members’ opinion if they believe women with genital schistosomiasis have a higher risk of HIV infection and infertility problems.*

10. **Would you prefer praziquantel be given only to school children or to the entire community?**

- *Inquire about FGD members’ opinion on community-wide delivery of PZQ. Emphasize on the benefits, fear of side effects.*

II. **Adult males ONLY**
1. **What is schistosomiasis?**

- *Ask about the local name(s) for schistosomiasis in the community.*
- *Ask members if they or any family member have ever suffered from schistosomiasis.*

2. **How do you acquire schistosomiasis?**

- *Ask FGD members how schistosomiasis is transmitted.*
- *Ask FGD members about their knowledge of the different forms of schistosomiasis.*
- *Ask members for their views on whether schistosomiasis can affect the reproductive system.*

3. **What are the common symptoms of schistosomiasis?**

- *Ask how to know people who have schistosomiasis in the community.*
- *Ask if schistosomiasis affects children, adult women, and men to the same extent, and what symptoms are associated with the disease.*
- *Ask FGD members if they consider schistosomiasis to be a fatal disease.*

4. **How do you treat schistosomiasis?**

- *Inquire from FGD members about how adults with schistosomiasis access treatment, focusing on (i) community-directed treatment provided by community health workers (CHWs) and (ii) the use of herbal concoctions.*
- *Should the PZQ drug for schistosomiasis be administered exclusively to school children or to everyone in the community?*

5. **Do you engage in contact with rivers or streams?**

- *Ask FGD members about river contact frequency.*
- *Inquire about the reason for visiting rivers or streams.*

6. **What is female genital schistosomiasis?**

- *Ask adult male FGD members if they know about FGS.*
- *Where did you obtain the information about FGS?*

7. **What is the cause of FGS?**

- *Explore their opinion on what the causes of FGS*
- *Inquire their opinions on whether FGS can be spread through sexual intercourse.*
- *Inquire whether members believe there is a connection between FGS and utilization of community rivers.*

8. **How severe do you think FGS is?**

- *Ask members whether they consider genital symptoms associated with schistosomiasis as a serious health condition.*
- *Probe members’ opinion if they believe women with genital schistosomiasis have a higher risk of HIV.*
- *Probe members’ opinion if they believe women with genital schistosomiasis have a higher risk of HPV infection and cervical cancer.*

**This concludes our discussion. Thank you for your valuable time today. If you have any further comments or questions, please feel free to share them with us.**

**End Time**: ______ Hours

# **KEY INFORMANT INTERVIEW (KII) GUIDE**

# I. **KEY COMMUNITY MEMBER**

A. **Introduction**

- *Please tell me about your designation in this community.*
- *What are your primary duties?*

**Now, I would like to ask you some specific questions about the topic of our discussion:**

1. **In your own words, what is schistosomiasis?**

- *What is it called by the local inhabitants in this community?*
- *How do people get schistosomiasis?*
- *What is the common symptom of inhabitants who have schistosomiasis?*
- *Are there different types of schistosomiasis? If yes, please explain.*
- *How often do local inhabitants use rivers or streams, and for what purposes?*

2. **How do people who suffer from schistosomiasis get treatment?**

- *Which age group is most affected by schistosomiasis in this community?*
- *Which gender is most affected by schistosomiasis in this community?*
- *Do individuals affected by schistosomiasis seek treatment at health centres, or do they pursue alternative treatment options?*
- *Does this community benefit from schistosomiasis treatment programs?*
- *If YES, is this intervention implemented through school-based programs or as community-wide treatment?*
- *Should PZQ be given just to schoolchildren or to the whole community?*

3. **What is female genital schistosomiasis (FGS)?**

- *Do you know of FGS? If yes, where did you hear about FGS?*
- *Can genital symptoms be due to schistosomiasis?*
- *Do you think abnormal genital symptoms in girls and women can be caused by visiting the rivers for domestic activities?*

4. **What is the treatment for females experiencing genital symptoms?**

- *What is the typical clinical diagnosis for females with genital abnormalities?*
- *What medication is commonly prescribed to females who experience abnormal genital symptoms?*
- *Does the community health centre offer services for females presenting with abnormal genital symptoms?*

5. **Assess opinions on the link between genital schistosomiasis and sexually transmitted infection.**

- *Is genital schistosomiasis a sexually transmitted disease?*
- *In your opinion, do girls/women with schistosomiasis face a higher risk of HIV infection?*
- *In your opinion, do girls or women with schistosomiasis have a higher risk of HPV infection or cervical cancer?*
- *In your opinion, do girls or women with schistosomiasis have a higher risk of infertility?*
- *Does this health facility offer sexual and reproductive health services (e.g training, diagnosis, health information, counselling, medical supplies, treatment)*

**Thank you so much for your time today. Do you have any additional thoughts or questions you would like to share with me?**

**End Time**: ______ Hours

# II. **COMMUNITY HEALTH PROFESSIONALS**

A. **Introduction**

- *Please tell me about your designation in this health facility.*
- *What are your primary duties?*

**Now, I would like to ask you some specific questions about the topic of our discussion:**

1. **In your own words, what is schistosomiasis?**
- *What is it called by the local inhabitants in this community?*
- *How do people get schistosomiasis?*
- *What is the common symptom of inhabitants who have schistosomiasis?*
- *How is schistosomiasis diagnosed at this health centre?*
- *Are there different forms of schistosomiasis? If yes, please explain*
- *How often do local inhabitants use rivers or streams, and for what purposes?*
  2. **How do you treat people who suffer from schistosomiasis?**
- *Which age group is most affected by schistosomiasis in this community?*
- *Which gender is most affected by schistosomiasis in this community?*
- *Which gender most frequently seeks treatment for schistosomiasis?*
- *How is schistosomiasis treated in the health facility?*
- *How is schistosomiasis treated in the community?*
  3. **What is female genital schistosomiasis (FGS)?**
- *Do you know of FGS? If yes, where did you hear about FGS?*
- *What genital symptoms are common among girls and women in this community?*
- *Do you think abnormal genital symptoms in girls and women can be caused by visiting the rivers for domestic activities?*
- *Can genital symptoms be due to schistosomiasis?*
  4. **What is the treatment for females experiencing genital symptoms?**
- *What is the typical clinical diagnosis for females with genital abnormalities?*
- *Do you consider schistosomiasis as a possible clinical diagnosis when girls or women present with abnormal genital manifestations in the health facility consultation?*
- *Do you know how to diagnose female genital schistosomiasis for girls/women experiencing abnormal genital symptoms?*
- *What treatment options do you provide for abnormal genital symptoms?*
- *Are there any services provided for addressing FGS in this health centre?*
  5. **Assess opinions on the link between genital schistosomiasis and sexually transmitted infection.**
- *Is genital schistosomiasis a sexually transmitted disease?*
- *In your opinion, do girls/women with schistosomiasis face a higher risk of HIV infection?*
- *In your opinion, do girls or women with schistosomiasis have a higher risk of HPV infection or cervical cancer?*
- *In your opinion, do girls or women with schistosomiasis have a higher risk of infertility?*
- *Does this health facility offer sexual and reproductive health services (e.g training, diagnosis, health information, counselling, medical supplies, treatment)*
- *Have you received any specific training on SRH services?*
- *If YES, how involved are you in the delivery of SRH services?*

6. **Opinion on implementation of schistosomiasis control strategy**

- *During which season and month is praziquantel treatment administered?*
- *Are you directly involved in PZQ treatment campaign in this community?*
- *Would you recommend school-based or community-wide treatment for schistosomiasis?*

**Thank you so much for your time today. Do you have any additional thoughts or questions you would like to share with me?**

**End Time**: ______ Hours
